# Social and Functional Risk Patterns and All-Cause Mortality Among US Adults with Chronic Liver Disease

**DOI:** 10.3390/medsci14040455

**Published:** 2026-08-04

**Authors:** Chukwuemeka E. Ogbu, Ndukwe J. Kalu, Henry E. Orjiudeh, Nonso Desmond Okeke, Maureen Okafor, Lekhya Kollu, Chinazor Umerah, Philip N. Okafor

**Affiliations:** 1 Department of Internal Medicine, Cape Fear Valley Health, Fayetteville, NC 28304, USA; 2 New York City Health + Hospitals/South Brooklyn Health, Brooklyn, NY 11235, USA; 3 Lewis Katz School of Medicine, Temple University, Philadelphia, PA 19140, USA; 4 Division of Gastroenterology and Hepatology, Mayo Clinic, Jacksonville, FL 32224, USA

**Keywords:** chronic liver disease, social determinants of health, functional limitation, latent class analysis, multiple imputation, all-cause mortality, health disparities

## Abstract

**Importance:** Chronic liver disease requires sustained engagement with the health system, which co-occurring social and functional risks may disrupt. Whether these risks form reproducible patterns with different mortality associations is uncertain. **Objective:** To identify social and functional risk patterns among US adults with chronic liver disease and evaluate their associations with all-cause mortality. **Design, Setting, and Participants:** Nationally representative cohort study of National Health Interview Survey sample adults interviewed from 2011 through 2018 and linked to the National Death Index through 31 December 2019. Participants were aged 18 years or older, eligible for mortality linkage, reported a chronic liver condition, and had no baseline liver cancer. **Exposures:** Latent class membership derived from 7 binary indicators: poverty, food insecurity, uninsurance, cost-related delayed care, no usual place of care, single-adult household status, and activity limitation. A complete 0- to 7-item count was a secondary exposure. **Main Outcomes and Measures:** All-cause mortality. Survey-weighted Cox models estimated hazard ratios (HRs) with sequential demographic, clinical, and liver-specific adjustment. Prespecified sensitivity analyses used 20 multiple imputed data sets, sampling-weighted pseudo-likelihood latent class models, complete-case re-estimation, a fixed 5-year horizon, and class-by-age interaction testing. **Results:** Among 3407 adults (weighted mean age, 54.0 years; 49.4% female), 529 deaths occurred over 17,297.6 person-years (median follow-up, 5.0 years [IQR, 3.0–7.0]). Four classes were retained: low social and access burden (weighted prevalence, 63.1%); functional limitation with financial strain (13.2%); poverty, single-adult household, and functional limitation (15.0%); and uninsurance with major access barriers (8.7%). Compared with the low-burden class, the functional-limitation/financial-strain class had higher adjusted mortality (HR, 1.95; 95% CI, 1.34–2.82; *p* < 0.001); the other 2 classes did not differ significantly. Sampling-weighted latent class analysis after multiple imputation reproduced 97.2% of modal assignments, and the association persisted with attenuation (HR, 1.55; 95% CI, 1.08–2.22; *p* = 0.02). Each additional risk was associated with higher mortality (exact count: HR, 1.24 [95% CI, 1.13–1.36]; imputed count: HR, 1.19 [95% CI, 1.10–1.29]). The class-by-age interaction was not significant (*p* = 0.41). **Conclusions and Relevance:** Among US adults with self-reported chronic liver disease, social and functional adversity was both cumulative and patterned. A profile combining functional limitation, food insecurity, and cost-related delayed care had the clearest independent mortality association despite largely retained insurance coverage. These profiles are not a validated clinical score, but the findings argue against reliance on any single indicator, particularly insurance status, as a marker of vulnerability.

## 1. Introduction

**Key Points**
**Question.** Do co-occurring social and functional risks form distinct patterns among US adults with chronic liver disease, and are those patterns differentially associated with all-cause mortality?
**Findings.** In this nationally representative cohort of 3407 adults with 529 deaths, latent class analysis identified 4 risk patterns. Compared with a low-burden class, a class defined by activity limitation, food insecurity, and cost-related delayed care, but almost no uninsurance, had higher adjusted mortality (hazard ratio, 1.95), an association that persisted in a sampling-weighted and multiple imputed sensitivity analysis (hazard ratio, 1.55). Cumulative risk burden was also associated with mortality.
**Meaning.** The configuration of social and functional adversity, not only its presence or count, carried prognostic information, suggesting that insurance status alone is an inadequate marker of vulnerability. External validation is required before clinical application.

Chronic liver disease (CLD) encompasses persistent hepatic injury from metabolic, alcohol-associated, viral, autoimmune, cholestatic, and genetic causes, spanning noncirrhotic fibrosis, compensated cirrhosis, and decompensation, and prognosis depends on etiology, fibrosis stage, portal-hypertension complications, and hepatic reserve [1,2]. Contemporary US guidance emphasizes etiology-specific recognition and treatment, including management of metabolic dysfunction-associated steatotic liver disease and curative therapy for hepatitis C [3,4]. Yet mortality from cirrhosis and liver cancer has risen in the United States [5], with disproportionate increases among younger adults and women [6], and these increases accumulated during a period of genuine therapeutic progress [5,7,8]. Clinical advances alone have not altered the trajectory of liver disease outcomes.

Clinical and behavioral factors remain central to risk assessment, but they do not fully explain who presents late, misses surveillance, cannot obtain medications, delays care, or dies. Social disadvantage shapes liver disease from development and diagnosis through decompensation, transplantation, and death [9,10]. Food insecurity has been associated with steatotic liver disease, advanced fibrosis, and mortality while cost-related barriers and transportation insecurity have been associated with poorer survival and with crisis-driven, rather than longitudinal care [11,12,13,14,15]. Living alone and social isolation may also confer risk, although household structure and functional social support are not interchangeable constructs [16,17].

Most prior work has examined one social factor at a time. That approach is incomplete because a patient with CLD is rarely only low-income, only uninsured, or only food insecure. The same person may live alone, have physical limitations, delay care because of cost, and lack a usual source of care, and these risks reinforce one another. Cumulative disadvantage is associated with mortality, but a count cannot distinguish patients with the same number of risks assembled in clinically different ways. For example, three risks might mean food insecurity, living alone, and activity limitation in one person, and uninsurance, no usual source of care, and cost-related delay in another [18,19]. Polysocial risk frameworks and latent class methods therefore seek to characterize both total burden and configuration [20,21], consistent with calls to connect screening to practical assistance rather than treating documentation as an end point [22].

Health-system context matters for interpretation. The United States has no single national insurance system, and substantial cost-sharing may persist despite nominal coverage [23]. European arrangements are heterogeneous rather than a single model. France uses near-universal statutory insurance with cost-sharing, whereas Spain operates a largely tax-funded service predominantly free at the point of care [24,25]. Consequently, an insurance-defined US class should not be assumed to map directly onto social vulnerability in European settings, where deprivation, support, functional barriers, and continuity of care may remain consequential despite universal coverage [26].

Pattern-based assessment may be especially relevant in CLD because care requires repeated laboratory testing, hepatocellular carcinoma (HCC) surveillance, antiviral or alcohol-use treatment, nutrition counseling, endoscopy, medication management, transplant evaluation, and timely recognition of decompensation [27]. Using the NHIS linked to the National Death Index (NDI), we sought to identify social and functional risk patterns among US adults with CLD and to evaluate the associations of these patterns, and of cumulative burden, with all-cause mortality.

## 2. Materials and Methods

### 2.1. Study Design and Data Source

We conducted a cohort study using the Integrated Public Use Microdata Series (IPUMS) version of the NHIS linked to mortality follow-up from the NDI. The NHIS is an annual household survey of the US civilian noninstitutionalized population using a stratified, multistage sampling design with clustering and design-based weights [28]. We used the 2019 public-use linked mortality file, which follows eligible respondents through 31 December 2019 [29]. The sample-adult mortality weight was divided by 8 for the pooled 2011–2018 analysis, consistent with IPUMS guidance [30]. Reporting follows the STROBE guidelines [31].

### 2.2. Study Population and Ethics

The analytic period was 2011 through 2018, selected to maximize the simultaneous availability of the 7 class indicators while preserving mortality follow-up. We restricted the sample to adults aged 18 years or older who were selected as the NHIS sample adult, were eligible for mortality linkage, and reported a history of chronic liver disease, defined as self-reported having ever had any chronic liver condition. Participants with baseline liver cancer were excluded from the primary analysis to avoid conflating chronic liver disease with prevalent liver malignancy at study entry.

CLD was operationalized using the NHIS variable LIVERCHRON, which identifies sample adults told by a health professional that they had cirrhosis, or another long-term liver condition. Before 2020, the questionnaire did not explicitly mention cirrhosis [32]. This definition is not clinical adjudication as it does not identify etiology, stage fibrosis, or distinguish compensated from decompensated disease.

This study used deidentified, publicly available NHIS files linked to mortality follow-up. The Cape Fear Valley Health Accreditation Council for Graduate Medical Education Institutional Review Board reviewed the study and determined that it did not constitute human-subject research and was therefore exempt, therefore informed consent was not required. Sex and race and ethnicity were ascertained from NHIS survey data and were included because social-risk exposure and mortality in CLD may differ across groups shaped by structural inequities.

### 2.3. Social and Functional Risk Indicators

Seven binary indicators were specified a priori: below-poverty-threshold status; low or very low family food security; no health insurance; delayed medical care because of cost during the prior 12 months; no usual place of medical care; being the only adult aged 18 years or older in the household; and any activity limitation. The activity-limitation measure summarizes limitations due to physical, mental, or emotional problems [33]. Because it can reflect both functional vulnerability and underlying illness, we refer to the latent constructs as social and functional risk patterns. Single-adult household status was treated as a proxy for potentially limited household support, not as a measure of loneliness or isolation.

Unknown, refused, and unascertained responses were coded as missing. At least 5 of 7 indicators were required for the primary cohort, and all otherwise eligible participants met this threshold. The cumulative count was calculated only after all 7 items were completed through multiple imputation. We also evaluated an exact complete-case 0-to-7-item count separately. A count summing available items was not retained because missing indicators would implicitly contribute zero, and the score would not be comparable across participants with 5, 6, or 7 observed items.

### 2.4. Model Selection for Primary Latent Class Analysis

We fit 2- through 6-class models using poLCA, retaining participants with partially observed manifest indicators by setting na.rm = FALSE [34]. Each model used 100 random starts and up to 10,000 iterations. We compared the log-likelihood, Akaike information criterion, Bayesian information criterion (BIC), minimum modal class size, mean maximum posterior probability, normalized entropy, replication of the best log-likelihood, substantive separation, and interpretability in accordance with class-enumeration guidance [35]. The prespecified rule was to select the lowest-BIC solution among models in which the smallest modal class comprised at least 5% of participants. Participants were assigned to the class with the highest posterior probability, and classes were ordered by increasing average observed burden and given descriptive labels.

### 2.5. Primary Latent Class Analysis

poLCA does not accommodate complex survey weights, and class derivation performed without weights may not generalize to the target population [36,37]. We therefore conducted a prespecified robustness analysis combining multiple imputation with sampling-weighted class estimation.

We generated 20 imputed data sets over 20 chained-equation iterations using mice [38]. Binary variables were imputed by logistic regression, unordered categorical variables by multinomial logistic regression, and body mass index by predictive mean matching. The imputation model included the 7 indicators, model covariates, survey year, log sampling weight, mortality status, and the Nelson-Aalen cumulative hazard [39]. Convergence was assessed using trace plots, potential scale-reduction factors, lag-1 autocorrelation, and logged events.

Within each imputed data set, the 7 responses were aggregated into observed patterns and latent class models were fit in randomLCA using normalized, nonnegative mortality weights as pattern frequencies [40]. Because sampling weights are not literal cell counts, and because the procedure does not incorporate NHIS strata or primary sampling units into latent-class variance estimation, we describe this throughout as a sampling-weighted pseudo-likelihood analysis rather than a design-based one, and treat its information criteria as descriptive fit diagnostics. We fit 2- through 6-class weighted models in the first 5 imputations and then fit the prespecified 4-class model in all 20, aligning class labels to the primary solution by minimizing squared distance across the 28 class-by-indicator cells. Modal-class Cox coefficients and covariance matrices were combined using Rubin rules [41].

### 2.6. Outcome and Covariates

The outcome was all-cause mortality as cause-specific death is limited in the public-use file. Follow-up was approximated from the interview year to the death year and quarter, because exact interview dates were unavailable and survivors were censored on 31 December 2019.

Models adjusted for age, sex, race and ethnicity (Hispanic, non-Hispanic White, non-Hispanic Black, or non-Hispanic other), education, census region, smoking status, alcohol use, body mass index, diabetes, hypertension, prior myocardial infarction, and any cancer. A liver-specific model added hepatitis history and a liver condition during the prior 12 months.

### 2.7. Statistical Analysis

Descriptive, survival, and regression analyses incorporated NHIS strata, primary sampling units, and the pooled sample-adult mortality weight [42]. Baseline characteristics are presented as unweighted counts with survey-weighted column percentages or survey-weighted means. Survey-weighted Cox proportional hazards models estimated unadjusted, adjusted, and liver-adjusted associations of class membership with mortality. A parallel model evaluated the cumulative count. Crude unweighted mortality rates per 1000 person-years and unadjusted survey-weighted survival curves were calculated for visualization.

Prespecified sensitivity analyses included a 3-class solution, complete-case re-estimation of classes and the exact count, and a fixed-horizon 5-year analysis among participants interviewed from 2011 through 2014 using survey-weighted quasi-Poisson regression with a log link. We also tested a global class-by-age interaction with age centered at 55 years and scaled per 10-year increment, using survey Wald test and summarized class-specific hazard ratios at ages 40, 55, and 70 years. Tests were 2-sided, with *p* < 0.05 considered significant. Analyses used R version 4.4.2 (R Foundation for Statistical Computing), using packages poLCA 1.6.0.1, survey 4.4-2, survival 3.8-3, mice 3.17.0, mitools 2.4, and randomLCA 1.1-4.

## 3. Results

### 3.1. Cohort and Completeness of Risk Data

Among 778,500 NHIS person records from 2011 through 2018, 257,652 were sample adults, 252,968 were eligible for mortality linkage, and 3531 reported a chronic liver condition. After excluding 124 participants with baseline liver cancer or unknown baseline liver cancer status, the primary cohort included 3407 participants (Table S1).

The requirement that at least 5 of 7 indicators be observed excluded no participants: 3235 (95.0%) had all 7 observed, 170 (5.0%) had 6, and 2 (0.1%) had 5. Missingness was driven almost entirely by below-poverty-threshold status (165 [4.8%]) Every other indicator was missing for 2 or fewer participants, and the dominant pattern was isolated missingness of poverty status (163 participants) (Tables S1 and S2).

The weighted mean age was 54.0 years; 49.4% were female, and 68.3% were non-Hispanic White. Overall, 20.7% were below the poverty threshold, 21.7% reported food insecurity, 10.6% were uninsured, 17.9% delayed care because of cost, 7.5% had no usual place of care, 27.3% were the only adult in the household, and 45.6% reported activity limitation (Table 1).

### 3.2. Class Enumeration and Profile Characteristics

The 5-class model had the lowest BIC (22,811.8), but its smallest modal class contained 3.5% of participants, below the prespecified 5% criterion, and the 6-class smallest class was 2.9%. Optimization stability also deteriorated as classes were added. The best log-likelihood was reproduced by 63 of 100 random starts for the 4-class model, but by 27 of 100 and 2 of 100 for the 5- and 6-class models. The 4-class model was retained because it satisfied the class-size criterion, achieved strong classification (mean maximum posterior probability, 0.880; normalized entropy, 0.771), and was substantively interpretable (Table 2).

The retained classes were low social and access burden (class 1; weighted prevalence, 63.1%), functional limitation with financial strain (class 2; 13.2%), poverty, single-adult household, and functional limitation (class 3; 15.0%), and uninsurance with major access barriers (class 4; 8.7%). Class 2 had high conditional probabilities of activity limitation (0.816) and food insecurity (0.553) with a 0.404 probability of cost-related delay, but essentially no uninsurance (0.010) and no poverty (0.010). Class 3 was characterized by poverty (0.999), only single-adult household status (0.768), and activity limitation (0.851). Class 4 was marked by uninsurance (0.873), cost-related delay (0.645), and no usual place for care (0.425), with comparatively low activity limitation (0.341) (Table 3).

### 3.3. All-Cause Mortality

**Figure 1:**
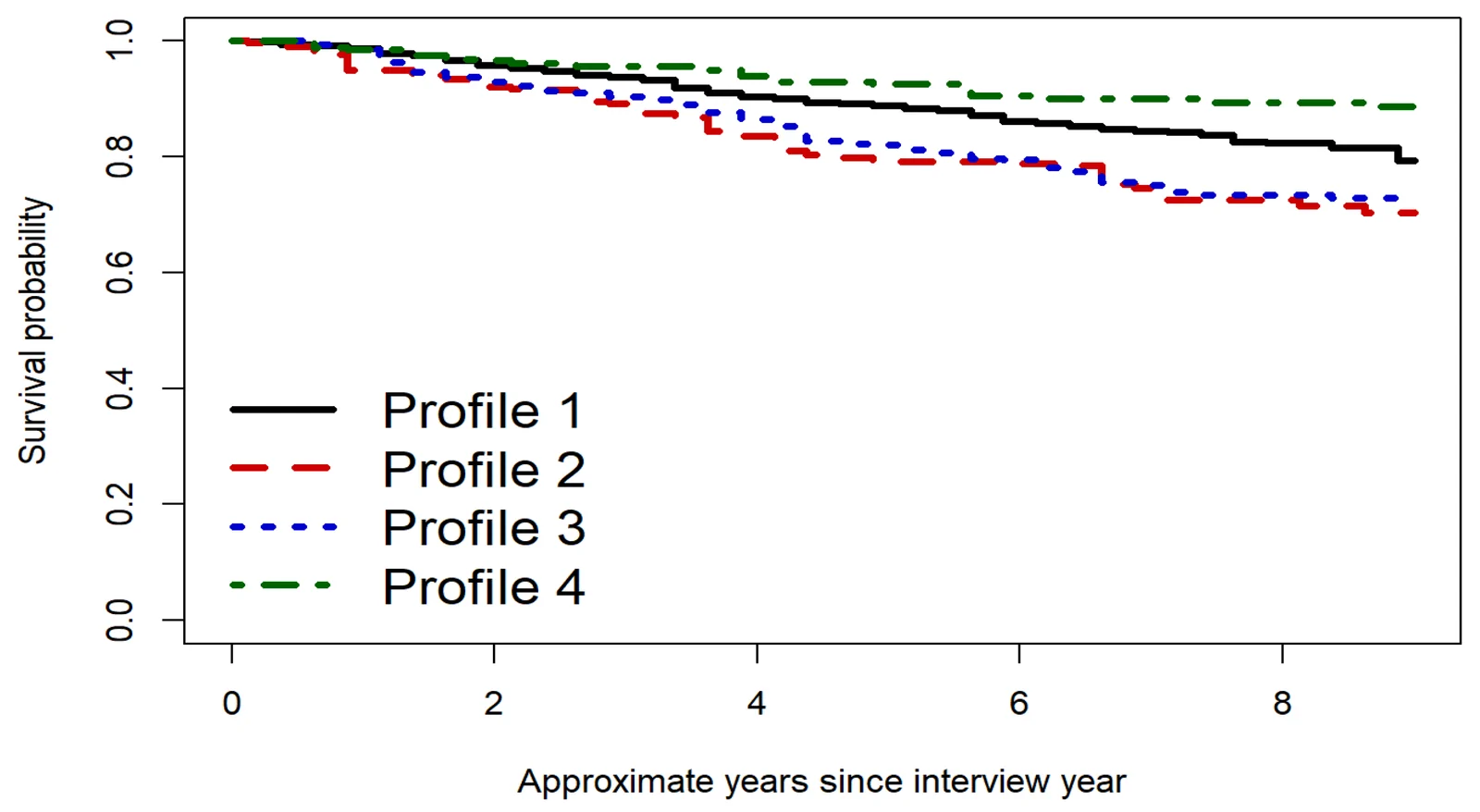
Unadjusted survey-weighted all-cause survival by social and functional risk class among US adults with chronic liver disease. Survey-weighted survival curves show the estimated probability of survival from the NHIS interview year through mortality follow-up by latent social-risk class. Class 1 denotes low social and access burden; class 2, functionally limited with financial strain; class 3, poor, isolated, and functionally limited; and class 4, uninsured with major access barriers. The analytic sample included US adults aged 18 years or older with chronic liver disease who were eligible for linkage to the National Death Index and had no baseline liver cancer.

During a median follow-up of 5.0 years (IQR, 3.0–7.0), 529 deaths occurred over 17,297.6 person-years. Crude mortality rates per 1000 person-years were 28.7 in class 1, 38.0 in class 2, 39.4 in class 3, and 14.3 in class 4. Survey-weighted survival curves by class are shown in Figure 1.

Median follow-up was longer in class 4 (7.0 years; IQR, 4.0–8.0) than in other classes, reflecting an earlier average interview year, so its low crude rate is not attributable to shorter observation (Table S3). Unadjusted survey-weighted survival was highest in class 4, which was also significantly younger (weighted mean age, 44.6 vs. 54.0–55.2 years).

Compared with class 1, class 2 had higher mortality in the unadjusted model (HR, 1.72; 95% CI, 1.21–2.45; *p* = 0.002), the adjusted model (HR, 1.95; 95% CI, 1.34–2.82; *p* < 0.001), and the liver-adjusted model (HR, 1.97; 95% CI, 1.36–2.85; *p* < 0.001). Class 3 was associated with mortality before adjustment (HR, 1.56; 95% CI, 1.20–2.03; *p* < 0.001) but not after (HR, 1.24; 95% CI, 0.90–1.70; *p* = 0.19). Class 4 had lower unadjusted mortality (HR, 0.60; 95% CI, 0.37–0.97; *p* = 0.04), with a null association after adjustment (HR, 1.15; 95% CI, 0.65–2.04; *p* = 0.63) (Table 4).

In the sampling-weighted, multiply imputed analysis, the class 2 association attenuated but remained significant (adjusted HR, 1.55; 95% CI, 1.08–2.22; *p* = 0.02; liver-adjusted HR, 1.57; 95% CI, 1.10–2.24; *p* = 0.01); classes 3 and 4 were null, and fractions of missing information were low (0.8–7.2%). Each additional risk was associated with higher mortality in both the imputed count (adjusted HR, 1.19; 95% CI, 1.10–1.29; *p* < 0.001) and the exact complete-case count (adjusted HR, 1.24; 95% CI, 1.13–1.36; *p* < 0.001) (Table 4 and Table S10).

### 3.4. Sampling-Weighted and Multiple Imputed Class Robustness

Twenty imputed data sets were generated. Final potential scale-reduction factors ranged from 0.986 to 1.024 and maximum absolute lag-1 autocorrelation was 0.100 (Table S12). In the weighted enumeration, 5-class models again attained the lowest pseudo-BIC but produced model-implied classes of 2.6% to 3.7%, whereas the 4-class solution maintained a smallest class of approximately 9.1% with higher entropy (Table S7).

Across all 20 imputations, exact modal agreement with the primary classes averaged 97.2% (range, 97.0–97.4%), and 96.8% of participants remained in the same weighted modal class in every imputation. Weighted modal prevalence was 61.9%, 14.2%, 15.4%, and 8.5% for classes 1 through 4, closely tracking primary values. The root-mean-square difference in conditional probabilities across the 28 cells was 0.064 (Tables S8 and S9).

### 3.5. Class-by-Age Interaction and Additional Sensitivity Analyses

The global class-by-age interaction was not significant (survey Wald F = 0.97; df = 3, 660; *p* = 0.41), providing no evidence that class associations differed by age. They showed a gradient for class 2, from 1.60 (95% CI, 0.64–4.05) at age 40 years to 2.14 (95% CI, 1.32–3.49) at age 70 years, and no class 4 association at any age (Table S11).

Class 2 remained associated with mortality in the complete-case–cohort (n = 3235; 495 deaths; adjusted HR, 1.84; 95% CI, 1.26–2.70; *p* = 0.002) (Table S6) and in the fixed-horizon 5-year analysis (adjusted risk ratio, 2.30; 95% CI, 1.60–3.31; *p* < 0.001), whereas classes 3 and 4 did not (Table S4). The 3-class solution retained an adjusted association for its intermediate-burden class (HR, 1.50; 95% CI, 1.11–2.02; *p* = 0.008) (Table S5).

## 4. Discussion

In this nationally representative cohort of US adults with self-reported CLD, four social and functional risk classes were not interchangeable in their relationship to mortality. The central finding was not that adversity mattered, but that its configuration mattered. Cumulative burden was associated with mortality in a graded fashion, yet the clearest independent excess risk was concentrated in the class marked by activity limitation, food insecurity, and cost-related delay despite largely retained insurance coverage. The class defined by poverty, single-adult household status, and limitation lost significance after adjustment, and the younger uninsurance/access-barrier class had no distinct association with mortality once baseline differences were accounted for. This pattern was stable across the three-class, complete-case, fixed-horizon, and sampling-weighted, multiply imputed analyses.

The weighted and imputed analysis reproduced approximately 97% of primary modal assignments, shifted class prevalence by no more than 1.2 percentage points, and left conditional probabilities substantively recognizable, indicating that the four-profile structure was not an artifact of unweighted derivation or item missingness. The class 2 estimate nevertheless attenuated from 1.95 to 1.55, which is worth stating plainly rather than minimizing. The weighted estimate is the more design-faithful of the two, and the excess mortality in this class is better regarded as on the order of 50% to 95% than as fixed at the primary value. Direction, significance, and interpretation were concordant.

The class most strongly associated with mortality is clinically recognizable: patients with high levels of functional limitation and food insecurity and frequent cost-related delay, but a model-based probability of uninsurance near zero. The pattern suggests that the effective use of care matters more than nominal coverage. Liver disease care depends on continuity, such as repeated visits, laboratory monitoring, imaging surveillance, medication access, and timely recognition of decompensation, and national data continue to document wide gaps in HCC surveillance, including among insured patients, that are unlikely to be random among those carrying several practical barriers to sustained engagement [43,44]. Coordination of care, multidisciplinary models, and telehealth are associated with better continuity or outcomes in cirrhosis [45,46].

Activity limitation may represent both a functional barrier and a marker of underlying severity. Disability and impaired physical function are associated with hospitalization, loss of independence, and mortality in advanced liver disease, and instruments such as the Liver Frailty Index were developed because this vulnerability is not captured by conventional severity scores [47,48,49]. These findings add that functional vulnerability appeared especially consequential when it coexisted with financial strain and food insecurity, which may explain why class 2 retained its association after adjustment, while class 3 attenuated. The results align with work on social frailty, in which social vulnerability carries prognostic information beyond traditional clinical indicators [50]. In CLD, the boundary between physical and social frailty may be more porous than usually acknowledged.

The behavior of class 4 is the most striking feature of the survival curves and warrants detailed treatment. The unadjusted advantage (HR, 0.60) disappeared entirely after adjustment (HR, 1.15), a pattern characteristic of confounding rather than protection. The largest contributor is age. Class 4 members were on average more than 10 years younger than every other class (44.6 vs. 54.0–55.2 years) and carried lower burdens of functional limitation, diabetes, hypertension, and prior myocardial infarction. This is the profile of a group in whom mortality has been deferred rather than averted, consistent with a compression-of-morbidity interpretation [51]. It is also consistent with the structure of US insurance. Because Medicare eligibility begins at age 65 years, an uninsured class in any US cross-sectional survey will be younger almost by construction [23].

Survival bias is harder to exclude, and we do not claim to have excluded it. Adults who report a diagnosed liver condition despite lacking insurance and a usual source of care may be a selected group who survived long enough to be diagnosed and interviewed, a form of index-event selection that can attenuate or invert associations among prevalent cases [52]. Two observations bear on this. The median follow-up in class 4 was longer, not shorter, than in other classes, so the null result is not an artifact of insufficient observation. Second, the global class-by-age interaction was not significant. The evidence favors confounding by baseline age and health as the dominant explanation, with survival bias plausible but unquantifiable in our study.

Whichever mechanism predominates, the absence of an adjusted mortality association should not be mistaken for an absence of clinical vulnerability. The consequences of this profile may be expressed less through near-term all-cause mortality than through delayed diagnosis, missed surveillance, and recurrent acute care, consistent with studies linking fragmented care and disability to readmission in cirrhosis [53]. A cohort followed from a younger age for two or three decades would be required to determine whether deferred risk in this group is ultimately realized.

European studies conducted under near-universal coverage establish that these associations are not an artifact of the US coverage gap. In a Swedish cirrhosis cohort, lower occupational skill was associated with poorer survival after adjustment for etiology, Model for End-Stage Liver Disease (MELD) score, Child-Pugh class, and comorbidity [54]. In Denmark, socioeconomic gradients were evident in the incidence of alcohol-related liver disease, and low functional social support, but not living alone itself, was associated with mortality, underscoring that household structure is not equivalent to effective support [55,56]. Across 9 European urban areas, inequalities in CLD and cirrhosis mortality were geographically persistent [57], and an Italian cohort in a universal-coverage setting found material deprivation to be associated with poorer follow-up, more decompensation events requiring hospitalization, and shorter survival [58]. Universal coverage can mitigate uninsurance but does not erase socially patterned barriers to longitudinal liver care.

The latent classes should not be used as a validated bedside prediction score, but they support several practical inferences. First, screening should be grouped rather than isolated. These results argue against using any single social risk, especially insurance status, as a proxy for vulnerability. An insured patient who is functionally limited, food insecure, and delaying care because of cost may face a higher mortality risk than a younger uninsured patient who is less medically fragile. Clinics caring for patients with CLD could administer a brief structured instrument covering, at minimum, food security, cost-related nonuse of care, functional status, and household support. Validated short tools make this feasible within existing workflows [59].

Second, a positive screen should trigger a defined referral pathway rather than documentation alone. Evidence that social-needs interventions improve outcomes remains heterogeneous, but the most promising signals come from interventions matched to the identified need. For food insecurity in chronic disease, medically tailored meal programs have been associated with reduced health care use [60,61]. Pathways for the constellation identified here would plausibly include a dietitian assessment and nutrition-program enrollment, pharmacy or financial navigation, therapy assessment for functional limitation, and social work evaluation. Third, care coordination should be intensified for patients with clustered social burden, not only for those with the most advanced disease, with attention to appointment outreach, consolidation of laboratory and imaging visits, transportation assistance, tracking of HCC surveillance completion rather than just ordering [43,44,45]. Fourth, functional limitation should be documented as a prognostic variable in its own right [48,49].

Several limitations should be weighed. Most consequentially, CLD was ascertained by self-report of a physician-diagnosed chronic liver condition, which agrees only moderately with medical records for frequently asymptomatic disease and is prone to under-ascertainment [62,63]. This single item cannot distinguish etiology, stage fibrosis, or separate compensated from decompensated cirrhosis. Because mortality risk differs by an order of magnitude across these strata, and because the social conditions defining the high-risk class may themselves be associated with more advanced disease at the time of interview, residual confounding by severity is a real possibility, and the observed patterns may partly reflect it. No severity measure, including MELD, is available in the public-use files [64], so this cannot be resolved analytically. Findings should therefore be read as describing risk patterns among community-dwelling adults reporting a chronic liver condition rather than as severity-adjusted prognostic estimates. Relatedly, activity limitation may itself be a consequence of advanced disease, multimorbidity, or frailty, raising the possibility of reverse causation.

Other limitations are more circumscribed. The hepatitis item cannot separate hepatitis B from C or capture direct-acting antiviral treatment, which became widely available during the study period and reduces mortality [65,66], so the hepatitis adjustment is coarse, and differential treatment access cannot be excluded. Alcohol use was classified only by status, without quantity, frequency, or binge episodes, making residual confounding likely given its dose–response relationship with liver-related mortality [67]. Single-adult household status is a proxy for limited support rather than a measure of isolation, and social risks were measured once and may have changed during follow-up. The prevalent cohort may be subject to survivor selection; follow-up was approximated from the interview quarter to the death quarter, and a median follow-up of 5.0 years may be insufficient to capture mortality from delayed diagnosis and missed surveillance. The outcome was all-cause rather than liver-specific. Finally, primary classes were derived without survey weights, and although the weighted analysis reproduced them, it did not use design-based, latent-class standard errors, and modal assignment with Rubin pooling does not fully propagate classification uncertainty [68]. Race and ethnicity categories were broad, and the observational design does not establish that modifying a class-defining risk would reduce mortality.

In terms of future directions, our analyses should be replicated in cohorts with objectively ascertained liver disease, etiologic classification, and fibrosis staging, so that risk patterns can be evaluated jointly with disease severity. Electronic health record cohorts linked to social-needs screening are well suited to this. Cause-specific mortality should be examined using restricted-use NDI files, and repeated measurements are needed because the persistence or resolution of adversity is likely more informative than a single baseline assessment. Methodologically, design-based mixture models and stepwise approaches that propagate classification uncertainty into survival outcomes would strengthen statistical inference. Intervention studies should test whether class-informed navigation improves surveillance, medication access, and survival, and a shorter clinical instrument should be developed only after external validation.

## 5. Conclusions

Among US adults with self-reported CLD, social and functional adversity was both cumulative and patterned, and those patterns were differentially associated with survival. A profile defined by activity limitation, food insecurity, and cost-related delayed care had the clearest independent mortality association, persisting when classes were re-estimated with sampling weights across multiple imputed data sets, whereas a younger uninsurance and access-barrier profile did not. The social conditions most relevant to prognosis in CLD are not captured by income or insurance alone, and risk stratification that accounts for clustered burden, particularly where it intersects with functional limitation, may better identify patients most likely to die despite advances in treatment. External validation is required before clinical application.

## Figures and Tables

**Table 1:** Baseline characteristics of US adults with chronic liver disease, overall and by social and functional risk class, NHIS 2011–2018.

Characteristic	Overall (*n* = 3407)	Class 1 (*n* = 1920)	Class 2 (*n* = 457)	Class 3 (*n* = 722)	Class 4 (*n* = 308)
Weighted class prevalence, %	100	63.1	13.2	15.0	8.7
Below poverty threshold	874 (20.7)	46 (2.8)	0 (0.0)	694 (100.0)	134 (42.1)
Food insecurity	830 (21.7)	2 (0.3)	353 (78.3)	351 (50.2)	124 (42.0)
Uninsured	365 (10.6)	59 (3.2)	0 (0.0)	9 (0.9)	297 (97.6)
Delayed care due to cost	666 (17.9)	95 (4.3)	243 (53.8)	114 (14.7)	214 (68.2)
No usual place for medical care	261 (7.5)	68 (3.5)	19 (3.8)	33 (4.9)	141 (46.0)
Only adult in household	1509 (27.3)	614 (18.7)	208 (26.6)	560 (64.1)	127 (27.7)
Any activity limitation	1722 (45.6)	636 (30.4)	377 (79.6)	605 (84.9)	104 (36.9)
Diabetes	834 (23.8)	432 (21.4)	144 (32.9)	211 (30.5)	47 (15.7)
Hypertension	1859 (53.4)	977 (50.0)	294 (65.0)	460 (65.5)	128 (40.2)
Prior myocardial infarction	304 (8.3)	131 (6.4)	57 (13.1)	101 (13.3)	15 (6.2)
Any cancer	595 (16.5)	353 (17.3)	90 (19.0)	123 (14.6)	29 (10.4)
Hepatitis history	1381 (38.9)	677 (34.5)	209 (47.0)	363 (48.4)	132 (42.4)
Recent liver condition	2185 (63.3)	1158 (61.2)	339 (71.5)	519 (71.1)	169 (52.7)
Female sex	1744 (49.4)	932 (47.3)	250 (54.6)	399 (52.0)	163 (52.6)
**Race and ethnicity**					
Hispanic	539 (16.2)	257 (14.8)	64 (14.9)	126 (15.7)	92 (29.4)
Non-Hispanic Black	359 (8.7)	126 (5.8)	59 (10.1)	142 (17.7)	32 (11.6)
Non-Hispanic other	240 (6.8)	141 (7.4)	23 (4.9)	47 (6.3)	29 (6.7)
Non-Hispanic White	2269 (68.3)	1396 (72.0)	311 (70.0)	407 (60.3)	155 (52.3)
**Education**					
High school	676 (18.7)	247 (12.6)	89 (19.7)	252 (34.9)	88 (33.2)
High school/GED	882 (25.2)	453 (23.0)	139 (29.5)	201 (28.9)	89 (28.1)
Some college/associate	1113 (32.9)	629 (33.3)	169 (37.5)	211 (27.4)	104 (32.3)
Bachelor’s or higher	722 (23.3)	583 (31.1)	58 (13.3)	55 (8.8)	26 (6.5)
**Census region**					
Northeast	550 (16.3)	320 (17.0)	70 (15.1)	135 (20.0)	25 (7.0)
Midwest	675 (20.5)	402 (21.6)	86 (17.3)	140 (20.8)	47 (16.6)
South	1129 (35.6)	580 (33.1)	176 (42.5)	256 (35.2)	117 (43.7)
West	1053 (27.7)	618 (28.4)	125 (25.1)	191 (24.0)	119 (32.7)
**Smoking status**					
Never	1398 (44.1)	904 (49.6)	138 (32.8)	238 (34.0)	118 (39.3)
Former	1061 (30.4)	681 (34.0)	141 (28.7)	187 (24.9)	52 (16.5)
Current	942 (25.4)	331 (16.4)	176 (38.5)	297 (41.1)	138 (44.2)
**Alcohol use**					
Lifetime abstainer	542 (17.0)	298 (16.6)	66 (16.5)	140 (20.7)	38 (14.2)
Former drinker	1071 (29.0)	526 (25.4)	178 (36.9)	280 (37.4)	87 (28.9)
Current drinker	1767 (54.0)	1076 (58.0)	210 (46.6)	300 (42.0)	181 (56.9)
Age, mean, y	54.0	54.9	54.0	55.2	44.6
Body mass index, mean	28.9	28.8	30.3	29.1	27.5
Social-risk count, mean	1.5	0.6	2.4	3.2	3.6

Data are presented as unweighted No. (survey-weighted column %) for categorical variables and survey-weighted means for continuous variables. Percentages were calculated among participants with non-missing data for the characteristic. Body mass index is calculated as weight in kilograms divided by height in meters squared. Latent classes were derived from below-poverty-threshold status, food insecurity, uninsurance, cost-related delayed care, no usual place for medical care, only adult-in-household status, and activity limitation. Class 1 denotes low social and access burden; class 2, functional limitation with financial strain; class 3, poverty, single-adult household, and functional limitation; and class 4, uninsurance with major access barriers. GED indicates General Educational Development; NHIS, National Health Interview Survey.

**Table 2:** Fit statistics for 2- through 6-class latent class models of social and functional risk.

No. of Classes	Log- Likelihood	AIC	BIC	Smallest Modal Class, %	Mean Maximum Posterior Probability	Normalized Entropy	Best Log- Likelihood Starts, No./100
2	−11,660.9	23,351.8	23,443.8	40.1	0.878	0.575	100
3	−11,343.5	22,733.0	22,874.1	9.5	0.870	0.701	100
**4**	**−11** **,** **281.5**	**22** **,** **625.1**	**22** **,** **815.2**	**9.0**	**0.880**	**0.771**	**63**
5	−11,247.3	22,572.6	22,811.8	3.5	0.873	0.782	27
6	−11,230.1	22,554.3	22,842.6	2.9	0.844	0.778	2

Models were fit without sampling weights using poLCA with 100 random starts and a maximum of 10,000 iterations. All 3407 participants were supplied to each model. 3235 had complete data on all 7 class indicators. Smallest class is based on modal class assignment. Higher mean maximum posterior probability and normalized entropy indicate better classification. The best-log-likelihood column gives the number of 100 random starts that reproduced the best log-likelihood within 1 × 10^−6^. The 4-class model was retained because it provided adequate class size, strong classification, greater optimization stability, and substantive interpretability. AIC indicates Akaike information criterion; BIC, Bayesian information criterion.

**Table 3:** Model-based conditional probabilities of social-risk indicators across latent classes.

Indicator	Class 1	Class 2	Class 3	Class 4
Below poverty threshold	0.054	0.010	0.999	0.417
Food insecurity	0.021	0.553	0.528	0.382
Uninsured	0.027	0.010	0.021	0.873
Delayed care due to cost	0.055	0.404	0.176	0.645
No usual place for medical care	0.038	0.031	0.042	0.425
Only adult in household	0.313	0.506	0.768	0.405
Any activity limitation	0.317	0.816	0.851	0.341

Values are model-based conditional probabilities from the 4-class latent class model. Higher values indicate a greater probability that members of a class endorsed the indicator. Class 1 denotes low social and access burden; class 2, functionally limited with financial strain; class 3, poor, isolated, and functionally limited; and class 4, uninsured with major access barriers.

**Table 4:** Associations of social and functional risk class and cumulative risk burden with all-cause mortality.

Exposure	Unadjusted HR (95% CI)	Adjusted HR (95% CI)	Adjusted + Liver Covariates HR (95% CI)
**Primary 4-class analysis**			
Class 2 vs. class 1	1.72 (1.21–2.45)	**1.95 (1.34–2.82)**	**1.97 (1.36–2.85)**
Class 3 vs. class 1	1.56 (1.20–2.03)	1.24 (0.90–1.70)	1.23 (0.89–1.70)
Class 4 vs. class 1	0.60 (0.37–0.97)	1.15 (0.65–2.04)	1.08 (0.60–1.95)
**Sampling-weighted, multiply imputed 4-class sensitivity**			
Class 2 vs. class 1	1.60 (1.13–2.28)	**1.55 (1.08–2.22)**	**1.57 (1.10–2.24)**
Class 3 vs. class 1	1.56 (1.19–2.04)	1.23 (0.90–1.69)	1.23 (0.90–1.69)
Class 4 vs. class 1	0.62 (0.38–1.01)	0.95 (0.55–1.64)	0.95 (0.55–1.64)
Per 1-point increase in multiply imputed count		**1.19 (1.10–1.29)**	**1.19 (1.10–1.29)**
**Exact complete-case count sensitivity**			
Per 1-point increase in exact 0- to 7-item count		**1.24 (1.13–1.36)**	**1.24 (1.13–1.36)**

Primary latent classes were derived with unweighted poLCA; primary outcome models incorporated NHIS sampling weights, strata, and primary sampling units. Adjusted models included age, sex, race and ethnicity, education, census region, smoking status, alcohol use, body mass index, diabetes, hypertension, prior myocardial infarction, and any cancer. Liver-adjusted models additionally included hepatitis history and recent liver condition. The weighted, multiple imputed sensitivity analysis used 20 imputed data sets and sampling-weighted pseudo-likelihood latent class models based on normalized mortality weights; modal-class Cox coefficients were combined using Rubin rules. All weighted, multiply imputed outcome models included 3407 participants and 529 deaths. The multiply imputed count was a complete 0- to 7-item sum in each data set. The exact complete-case count required all 7 indicators to be observed (n = 3235; 495 deaths). A count computed with missing indicators implicitly treated as zero is not reported. HR indicates hazard ratio; NHIS, National Health Interview Survey.

## Data Availability

The NHIS public-use data and linked mortality files are publicly available from the National Center for Health Statistics and IPUMS NHIS. Statistical code and analytic documentation are available from the corresponding author on reasonable request.

## Supplementary Materials

The following supporting information can be downloaded at: https://www.mdpi.com/article/10.3390/medsci14040455/s1. Table S1. Participant Selection and Completeness of Social-Risk Indicators; Table S2. Detailed Missing-Data Patterns for the Seven Social-Risk Indicators; Table S3. Follow-up Time, Deaths, and Crude Mortality Rates by Primary Latent Social-Risk Class; Table S4. Five-Year Mortality Sensitivity Analysis by Primary Latent Social-Risk Class, Participants Interviewed 2011-2014; Table S5. Sensitivity Analysis Using the 3-Class Latent Class Solution; Table S6. Complete-Case Sensitivity Analysis Requiring All Seven Social-Risk Indicators Observed; Table S7. Sampling-Weighted Pseudo-Likelihood LCA Model-Selection Diagnostics Across Five Imputed Datasets; Table S8. Stability and Prevalence of the Sampling-Weighted, Multiply Imputed 4-Class Solution; Table S9. Mean Conditional Probabilities in the Sampling-Weighted, Multiply Imputed 4-Class Solution; Table S10. Pooled Survey-Weighted Cox Models After Multiple Imputation and Sampling-Weighted Latent Class Analysis; Table S11. Class-by-Age Interaction and Age-Specific Mortality Associations; Table S12. Multiple-Imputation Methods and Final Convergence Diagnostics.
