# Supplementary material for: Social and Functional Risk Patterns and All-Cause Mortality Among US Adults with Chronic Liver Disease
Source: Med Sci (Basel). 2026 Aug 4;14(4):455. doi: 10.3390/medsci14040455 (PMC13515290; doi:10.3390/medsci14040455)
Supplement: Supplementary file 1 [file medsci-14-00455-s001.zip › medsci-4377834-supplementary.pdf]

Table S1. Participant Selection and Completeness of Social-Risk Indicators

Panel A. Participant selection

Selection step	No. remaining	No. excluded from previous step
All NHIS person records, 2011-2018	778,500	0
Selected sample adults (ASTATFLG = 1)	257,652	520,848
Age 18 years or older	257,652	0
Eligible for linked mortality	252,968	4,684
Self-reported chronic liver condition	3,531	249,437
No baseline liver cancer and known liver-cancer status	3,407	124
At least 5 of 7 social-risk indicators observed (primary cohort)	3,407	0
Complete-case sensitivity subset: all 7 indicators observed	3,235	Not applicable
Participants with 5 or 6 indicators observed and retained in primary cohort	172	Not excluded

Panel B. Completeness of the seven indicators

No. of social-risk indicators observed	Unweighted No.	Unweighted %
5	2	0.1
6	170	5.0
7	3,235	95.0

Panel C. Item-level missingness in the primary cohort

Indicator	Observed, No.	Missing, No.	Missing, %
Below poverty threshold	3,242	165	4.84
Food insecurity	3,405	2	0.06
Uninsured	3,405	2	0.06
Delayed care due to cost	3,405	2	0.06
No usual place for medical care	3,406	1	0.03
Only adult in household	3,407	0	0.00
Any activity limitation	3,405	2	0.06

All 3407 participants in the primary cohort had at least five of seven indicators observed. Therefore, no participant was excluded for insufficient indicator data. The 3235 participants with all seven indicators observed formed a complete-case sensitivity subset. The 172 participants with five or six observed indicators remained in the primary analysis.

Table S2. Detailed Missing-Data Patterns for the Seven Social-Risk Indicators

Poverty	Food insecurity	Uninsured	Cost-related delay	No usual source	Only adult	Activity limitation	Unweighted No.	Unweighted %	Weighted %
Observed	Observed	Observed	Observed	Observed	Observed	Observed	3,235	94.95	94.79
Missing	Observed	Observed	Observed	Observed	Observed	Observed	163	4.78	5.04
Observed	Observed	Missing	Observed	Observed	Observed	Observed	2	0.06	0.02
Observed	Observed	Observed	Observed	Observed	Observed	Missing	2	0.06	0.03
Missing	Missing	Observed	Observed	Observed	Observed	Observed	1	0.03	0.06
Missing	Observed	Observed	Missing	Observed	Observed	Observed	1	0.03	0.03
Observed	Missing	Observed	Observed	Observed	Observed	Observed	1	0.03	0.00
Observed	Observed	Observed	Missing	Observed	Observed	Observed	1	0.03	0.01
Observed	Observed	Observed	Observed	Missing	Observed	Observed	1	0.03	0.01

Observed and missing statuses are shown in the following indicator order: below-poverty-threshold status, food insecurity, uninsurance, delayed care due to cost, no usual place for medical care, only adult in household, and activity limitation. Weighted percentages use the pooled sample-adult mortality weight. Percentages may not total exactly 100 because of rounding.

Table S3. Follow-up Time, Deaths, and Crude Mortality Rates by Primary Latent Social-Risk Class

Social-risk class	Unweighted No.	Deaths, No.	Person-years	Crude mortality rate per 1000 person-years	Median follow-up, y	IQR, y
Overall	3407	529	17,297.6	30.6	5.0	3.0-7.0
Class 1	1920	273	9,523.0	28.7	5.0	3.0-7.0
Class 2	457	85	2,239.0	38.0	4.9	3.0-7.0
Class 3	722	144	3,652.0	39.4	5.0	3.0-7.0
Class 4	308	27	1,884.0	14.3	7.0	4.0-8.0

Crude mortality rates were calculated from unweighted deaths divided by unweighted person-years. Follow-up was approximated from interview year to death or censoring through December 31, 2019. IQR indicates interquartile range.

Table S4. Five-Year Mortality Sensitivity Analysis by Primary Latent Social-Risk Class, Participants Interviewed 2011-2014

Comparison	Adjusted RR	95% CI	P value
Class 2 vs class 1	2.30	1.60-3.31	<.001
Class 3 vs class 1	1.02	0.67-1.57	.92
Class 4 vs class 1	1.17	0.63-2.17	.63

Results are from a survey-weighted fixed-horizon 5-year mortality model using quasi-Poisson regression with a log link among participants interviewed in 2011-2014. Class 1 was the reference group. The model adjusted for age, sex, race and ethnicity, education, region, smoking status, alcohol use, body mass index, diabetes, hypertension, prior myocardial infarction, and any cancer. CI indicates confidence interval; RR, risk ratio.

Table S5. Sensitivity Analysis Using the 3-Class Latent Class Solution

Comparison	Unadjusted HR (95% CI)	P value	Adjusted HR (95% CI)	P value	Adjusted + liver HR (95% CI)	P value
3-Class 2 vs 3-class 1	1.69 (1.33-2.15)	<.001	1.50 (1.11-2.02)	.008	1.49 (1.10-2.02)	.009
3-Class 3 vs 3-class 1	0.59 (0.37-0.94)	.028	1.06 (0.61-1.86)	.83	1.00 (0.57-1.78)	.99

Results are from survey-weighted Cox proportional hazards models using the 3-class latent class solution. The lowest-burden class served as the reference. Adjusted models included age, sex, race and ethnicity, education, region, smoking status, alcohol use, body mass index, diabetes, hypertension, prior myocardial infarction, and any cancer. The liver-adjusted model additionally included hepatitis history and recent liver condition. CI indicates confidence interval; HR, hazard ratio.

Table S6. Complete-Case Sensitivity Analysis Requiring All Seven Social-Risk Indicators Observed

Panel A. Latent class model selection

No. of classes	Log-likelihood	AIC	BIC	Smallest modal class, %	Mean maximum posterior probability
2	-11214.5	22459.0	22550.2	40.2	0.881
3	-10910.3	21866.6	22006.5	9.6	0.871
4	-10849.3	21760.6	21949.1	9.2	0.886

Panel B. Class size and exact social-risk burden

Class	Unweighted No.	Mean exact social-risk count
Class 1	1,811	0.81
Class 2	433	2.64
Class 3	694	3.32
Class 4	297	3.71

Panel C. All-cause mortality associations

Exposure	Unadjusted HR (95% CI)	P value	Adjusted HR (95% CI)	P value	Adjusted + liver HR (95% CI)	P value
Class 2 vs class 1	1.77 (1.22-2.58)	.003	1.84 (1.26-2.70)	.002	1.87 (1.27-2.74)	.001
Class 3 vs class 1	1.63 (1.25-2.14)	<.001	1.29 (0.93-1.80)	.128	1.29 (0.93-1.80)	.129
Class 4 vs class 1	0.65 (0.40-1.06)	.082	1.19 (0.66-2.12)	.565	1.12 (0.62-2.04)	.700
Per 1-point increase in exact count	-	-	1.24 (1.13-1.36)	<.001	-	-

The complete-case sample included 3235 participants and 495 deaths. Latent classes were independently re-estimated and ordered by increasing mean exact social-risk count. Class labels indicate this burden order and do not imply identical individual membership to the primary solution. Adjusted models included age, sex, race and ethnicity, education, region, smoking status, alcohol use, body mass index, diabetes, hypertension, prior myocardial infarction, and any cancer. Liver-adjusted models additionally included hepatitis history and recent liver condition. AIC indicates Akaike information criterion; BIC, Bayesian information criterion; CI, confidence interval; HR, hazard ratio.

Table S7. Sampling-Weighted Pseudo-Likelihood LCA Model-Selection Diagnostics Across Five Imputed Datasets

No. of classes	Mean pseudo-AIC (range)	Mean pseudo-BIC (range)	Smallest model-implied class, mean % (range)	Normalized entropy, mean (range)	Lowest pseudo-BIC, No./5 imputations
2	21866.4 (21843.7-21903.1)	21958.4 (21935.7-21995.1)	40.8 (40.3-41.8)	0.592 (0.587-0.596)	0
3	21353.1 (21327.5-21386.5)	21494.2 (21468.6-21527.6)	9.9 (9.8-10.0)	0.723 (0.720-0.726)	0
**4**	**21214.0 (21184.3-21247.4)**	**21404.1 (21374.5-21437.5)**	**9.1 (9.0-9.1)**	**0.795 (0.792-0.799)**	**0**
5	21149.7 (21117.6-21178.7)	21388.9 (21356.8-21417.9)	3.1 (2.6-3.7)	0.808 (0.803-0.814)	5
6	21130.9 (21101.7-21155.9)	21419.2 (21389.9-21444.2)	2.7 (2.0-3.6)	0.798 (0.687-0.838)	0

Each model used normalized nonnegative mortality sampling weights as response-pattern frequencies and was fit in the first five imputed datasets with 25 random starts. These are pseudo-likelihood fit criteria and do not incorporate NHIS strata and primary sampling units into latent-class standard errors. The 5-class model had the lowest pseudo-BIC in all five datasets but generated a smallest model-implied class of approximately 2.6%-3.7%. The prespecified 4-class solution was retained for the robustness analysis because it maintained a class size near 9%, strong entropy, interpretability, and close agreement with the primary classes. AIC indicates Akaike information criterion; BIC, Bayesian information criterion; LCA, latent class analysis; NHIS, National Health Interview Survey.

Table S8. Stability and Prevalence of the Sampling-Weighted, Multiply Imputed 4-Class Solution

Panel A. Class prevalence and assignment agreement

Class	Primary modal weighted prevalence, %	Weighted-MI modal prevalence, mean % (SD)	Weighted-MI model-implied probability, mean % (SD)	Agreement with primary class, %
Class 1	63.1	61.9 (0.1)	58.0 (0.4)	96.8
Class 2	13.2	14.2 (0.1)	18.6 (0.3)	98.1
Class 3	15.0	15.4 (0.1)	14.4 (0.2)	97.8
Class 4	8.7	8.5 (0.1)	9.1 (0.1)	96.6

Panel B. Overall stability diagnostics

Diagnostic	Result across 20 imputations
Overall exact agreement with primary modal class	97.2% (97.0%-97.4%)
Minimum model-implied class probability	9.1% (9.0%-9.2%)
Normalized entropy	0.794 (0.789-0.799)
Weighted pseudo-BIC	21399.5 (21355.0-21437.5)
Unique response patterns	121-122
Optimizer termination code	Code 1 in 20/20 fits
Conditional-probability alignment RMSE	0.064 (0.063-0.066)

Weighted-MI values summarize 20 imputed datasets. Modal weighted prevalence is based on the class with the highest posterior probability for each participant. Model-implied probability is the fitted latent-class proportion. Class-specific agreement is the proportion of primary-class assignments reproduced by the weighted-MI modal solution across all person-imputation records. Conditional-probability alignment RMSE was calculated across the 28 class-by-indicator cells. MI indicates multiple imputation; RMSE, root-mean-square error.

Table S9. Mean Conditional Probabilities in the Sampling-Weighted, Multiply Imputed 4-Class Solution

Indicator	Class 1	Class 2	Class 3	Class 4
Below poverty threshold	0.048 (0.003)	<0.001 (<0.001)	1.000 (0.000)	0.395 (0.010)
Food insecurity	0.009 (0.002)	0.522 (0.006)	0.539 (0.005)	0.406 (0.003)
Uninsured	0.030 (0.001)	<0.001 (<0.001)	0.016 (0.002)	0.955 (0.002)
Delayed care due to cost	0.037 (0.001)	0.406 (0.004)	0.163 (0.003)	0.642 (0.004)
No usual place for medical care	0.035 (0.000)	0.042 (0.001)	0.054 (0.001)	0.429 (0.002)
Only adult in household	0.177 (0.001)	0.309 (0.002)	0.622 (0.005)	0.266 (0.002)
Any activity limitation	0.286 (0.002)	0.738 (0.004)	0.832 (0.007)	0.369 (0.002)

Values are mean conditional probabilities (SD) across 20 imputed datasets after class alignment to the primary solution. Class 1 denotes low social and access burden; class 2, functional limitation with financial strain; class 3, poverty, single-adult household, and functional limitation; and class 4, uninsurance with major access barriers. MI indicates multiple imputation; SD, standard deviation.

Table S10. Pooled Survey-Weighted Cox Models After Multiple Imputation and Sampling-Weighted Latent Class Analysis

Model	Exposure	HR	95% CI	P value	Fraction missing information, %
Unadjusted class model	Class 2 vs class 1	1.60	1.13-2.28	.008	1.5
Unadjusted class model	Class 3 vs class 1	1.56	1.19-2.04	.001	3.8
Unadjusted class model	Class 4 vs class 1	0.62	0.38-1.01	.053	1.8
Adjusted class model	Class 2 vs class 1	1.55	1.08-2.22	.017	1.9
Adjusted class model	Class 3 vs class 1	1.23	0.90-1.69	.199	6.5
Adjusted class model	Class 4 vs class 1	0.95	0.55-1.64	.857	1.6
Adjusted + liver covariates class model	Class 2 vs class 1	1.57	1.10-2.24	.014	2.1
Adjusted + liver covariates class model	Class 3 vs class 1	1.23	0.90-1.69	.201	7.2
Adjusted + liver covariates class model	Class 4 vs class 1	0.95	0.55-1.64	.863	1.6
Adjusted count model	Per 1-point increase in imputed count	1.19	1.10-1.29	<.001	0.8
Adjusted + liver covariates count model	Per 1-point increase in imputed count	1.19	1.10-1.29	<.001	0.9

All models included 3407 participants and 529 deaths in each of 20 imputed datasets. Adjusted models included age, sex, race and ethnicity, education, region, smoking status, alcohol use, body mass index, diabetes, hypertension, prior myocardial infarction, and any cancer. Liver-adjusted models additionally included hepatitis history and recent liver condition. Rubin pooling captures imputation uncertainty in the fitted Cox coefficients but does not fully propagate latent-class estimation, model-selection, or classification uncertainty. CI indicates confidence interval; FMI, fraction of missing information; HR, hazard ratio.

Table S11. Class-by-Age Interaction and Age-Specific Mortality Associations

Panel A. Age and mortality by primary class

Class	Unweighted No.	Deaths, No.	Weighted mean age (SE), y	Weighted mortality proportion (SE), %
Class 1	1,920	273	54.9 (0.5)	11.7 (0.9)
Class 2	457	85	54.0 (0.9)	18.5 (2.7)
Class 3	722	144	55.2 (0.7)	17.9 (1.8)
Class 4	308	27	44.6 (0.9)	8.6 (1.9)

Panel B. Global interaction test

Test	F statistic	Numerator df	Denominator df	P value
Global class-by-age interaction	0.97	3	660	.408

Panel C. Age-specific class hazard ratios

Comparison	Age, y	HR	95% CI	P value
Class 2 vs Class 1	40	1.60	0.64-4.05	.318
Class 2 vs Class 1	55	1.85	1.18-2.90	.007
Class 2 vs Class 1	70	2.14	1.32-3.49	.002
Class 3 vs Class 1	40	1.87	0.92-3.78	.082
Class 3 vs Class 1	55	1.43	0.95-2.16	.089
Class 3 vs Class 1	70	1.09	0.77-1.54	.617
Class 4 vs Class 1	40	1.33	0.54-3.29	.536
Class 4 vs Class 1	55	1.15	0.65-2.03	.621
Class 4 vs Class 1	70	1.00	0.46-2.16	.999

The interaction model used age centered at 55 years and scaled per 10-year increase. The global interaction test was a survey Wald test. Age-specific hazard ratios were calculated from the interaction model at ages 40, 55, and 70 years. Because the global interaction was not statistically significant, age-specific estimates should be interpreted as descriptive rather than evidence of effect modification. CI indicates confidence interval; df, degrees of freedom; HR, hazard ratio; SE, standard error.

Table S12. Multiple-Imputation Methods and Final Convergence Diagnostics

Variable	Missing, No. (%)	Imputation method	Final lag-1 autocorrelation	Final PSRF
Below poverty threshold	165 (4.84)	Logistic regression	0.100	1.017
Food insecurity	2 (0.06)	Logistic regression	0.026	1.019
Uninsured	2 (0.06)	Logistic regression	0.052	0.986
Delayed care due to cost	2 (0.06)	Logistic regression	-0.004	1.010
No usual place for medical care	1 (0.03)	Logistic regression	-0.003	0.996
Any activity limitation	2 (0.06)	Logistic regression	0.000	1.013
Diabetes	5 (0.15)	Logistic regression	-0.019	1.008
Hypertension	11 (0.32)	Logistic regression	0.075	1.008
Prior myocardial infarction	8 (0.23)	Logistic regression	-0.061	1.005
Any cancer	6 (0.18)	Logistic regression	-0.018	1.001
Hepatitis history	27 (0.79)	Logistic regression	0.065	1.024
Recent liver condition	11 (0.32)	Logistic regression	-0.052	0.998
Fair or poor self-rated health	5 (0.15)	Logistic regression	-0.010	0.993
Educational attainment	14 (0.41)	Polytomous logistic regression	0.028	1.007
Smoking status	6 (0.18)	Polytomous logistic regression	0.074	1.010
Alcohol-use status	27 (0.79)	Polytomous logistic regression	0.004	1.006
Body mass index	335 (9.83)	Predictive mean matching	0.017	1.003

Twenty imputed datasets were generated over 20 iterations. The event indicator, Nelson-Aalen cumulative hazard, survey year, and log sampling weight were included as predictors. No MICE events were logged. Final diagnostics are from iteration 20; potential scale-reduction factors were 0.986-1.024 and the maximum absolute lag-1 autocorrelation was 0.100. MICE indicates multivariate imputation by chained equations; PMM, predictive mean matching; PSRF, potential scale-reduction factor.
